# Accumulation of the Auxin Precursor Indole-3-Acetamide Curtails Growth through the Repression of Ribosome-Biogenesis and Development-Related Transcriptional Networks

**DOI:** 10.3390/ijms22042040

**Published:** 2021-02-18

**Authors:** Beatriz Sánchez-Parra, Marta-Marina Pérez-Alonso, Paloma Ortiz-García, José Moya-Cuevas, Mathias Hentrich, Stephan Pollmann

**Affiliations:** 1 Institut für Biologie, Bereich Pflanzenwissenschaften, Karl-Franzens Universität Graz, 8010 Graz, Austria; beatriz.sanchez-parra@uni-graz.at; 2 Centro de Biotecnología y Genómica de Plantas, Universidad Politécnica de Madrid (UPM)–Instituto Nacional de Investigación y Tecnología Agraria Y Alimentación (INIA), Campus de Montegancedo, Pozuelo de Alarcón, 28223 Madrid, Spain; marta.perez.alonso@slu.se (M.-M.P.-A.); p.ortiz@upm.es (P.O.-G.); jose.moya@upm.es (J.M.-C.); 3 Umeå Plant Science Center, Umeå University, 90736 Umeå, Sweden; 4 Lehrstuhl für Pflanzenphysiologie, Ruhr-Universität Bochum, 44780 Bochum, Germany; mathias.hentrich@rub.de; 5 Departamento de Biotecnología-Biología Vegetal, Escuela Técnica Superior de Ingeniería Agronómica, Alimentaria y de Biosistemas, Universidad Politécnica de Madrid (UPM), 28040 Madrid, Spain

**Keywords:** *Arabidopsis thaliana*, indole-3-acetic acid, indole-3-acetamide, indole glucosinolate, seed maturation, seed size, ribosome biogenesis, plant growth

## Abstract

The major auxin, indole-3-acetic acid (IAA), is associated with a plethora of growth and developmental processes including embryo development, expansion growth, cambial activity, and the induction of lateral root growth. Accumulation of the auxin precursor indole-3-acetamide (IAM) induces stress related processes by stimulating abscisic acid (ABA) biosynthesis. How IAM signaling is controlled is, at present, unclear. Here, we characterize the *ami1* *rooty* double mutant, that we initially generated to study the metabolic and phenotypic consequences of a simultaneous genetic blockade of the indole glucosinolate and IAM pathways in *Arabidopsis* *thaliana*. Our mass spectrometric analyses of the mutant revealed that the combination of the two mutations is not sufficient to fully prevent the conversion of IAM to IAA. The detected strong accumulation of IAM was, however, recognized to substantially impair seed development. We further show by genome-wide expression studies that the double mutant is broadly affected in its translational capacity, and that a small number of plant growth regulating transcriptional circuits are repressed by the high IAM content in the seed. In accordance with the previously described growth reduction in response to elevated IAM levels, our data support the hypothesis that IAM is a growth repressing counterpart to IAA.

## 1. Introduction

**Figure 1:**
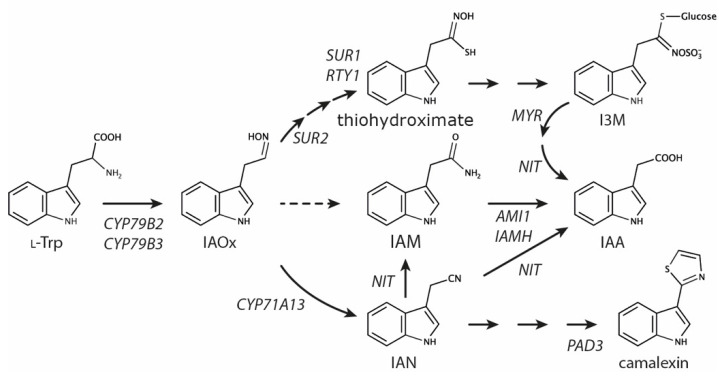
Metabolic pathways of the indole-3-acetaldoxime shunt in *Arabidopsis thaliana*. Schematic overview of the biosynthesis of l-Trp derived indole glucosinolate, camalexin, and indole-3-acetic acid. Dashed lines indicate reaction steps for which corresponding genes/enzymes have not yet been identified. *AMI1, AMIDASE1; CYP71A13, CYTOCHROME P450 MONOOXYGENASE 71A13, CYP79B2, CYTOCHROME P450 MONOOXYGENASE 79B2, CYP79B3, CYTOCHROME P450 MONOOXYGENASE 79B3,* I3M, glucobrassicin; IAA, indole-3-acetic acid; IAM, indole-3-acetamide; *IAMH*, *IAM HYDROLASE1-2*, IAN, indole-3-acetonitrile; IAOx, indole-3-acetaldoxime; l-Trp, l-tryptophan; *MYR*, *MYROSINASE*; *NIT*, *NITRILASE1-3*; *PAD3, PHYTOALEXIN DEFICIENT3* (*CYP71B15)*; *RTY, ROOTY*; *SUR1*, *SUPERROOT1*; *SUR2*, *SUPERROOT2 (CYP83B1)*.

The auxin indole-3-acetic acid (IAA) is a major endogenous growth factor in plants and is linked with a great variety of different developmental processes and adaptive responses, including elongation growth, polar development, cambial activity, gravitropism and phototropism, respectively. Thus, it is not surprising that IAA is recognized as an essential phytohormone necessary to ensure optimal plant growth and development [1,2]. The main source of auxin in plants is the indole-3-pyruvate (IPyA) pathway, encompassing tryptophan aminotransferases (TAA1/TAR2) and flavin containing monooxygenases (YUC1-11) that convert l-tryptophan (l-Trp) to IAA via the intermediate IPyA [3,4]. The spatio–temporal expression of these key players in auxin biosynthesis is tightly developmentally controlled [5]. Alongside the main IPyA pathway, plants are assumed to involve a small number of additional pathways in the formation of IAA. These pathways act either redundantly or in a parallel manner with the main route [6,7,8]. The Brassicaceae family, including the model plant *Arabidopsis thaliana*, possesses the additional indole-3-acetaldoxime (IAOx) pathway (Figure 1).

IAOx is considered to be an important metabolic branching point, involved in connecting primary and secondary metabolism [9,10,11]. Remarkably, IAOx serves as the common biochemical source for the formation of l-Trp derived glucosinolates [12], such as glucobrassicin, and camalexin [13], two important plant defense compounds in Arabidopsis. The formation of both metabolites is induced in response to biotic stresses and involves the transcription factors MYB34, MYB51, MYB122 and WRKY33 for controlling indole glucosinolate and camalexin biosynthesis, respectively [14,15]. The *cyp79b2 cyp79b3* double mutant, deficient in the formation of IAOx, shows a wild type-like phenotype and unaltered IAA levels under normal growth conditions [9,10,16], but demonstrates a significantly increased susceptibility towards pathogens [17], which underscores the importance of the IAOx pathway in biotic stress responses. Furthermore, *cyp79b2 cyp79b3* has been reported to contain drastically reduced indole-3-acetamide (IAM) contents, suggesting that a large proportion of IAM in Arabidopsis originates from IAOx [10]. IAM has, however, been found in several non-Brassica plant species that normally do not possess the IAOx pathway [18]. Hence, it must be assumed that there is an alternative biosynthetic route leading to IAM, possibly through yet unidentified tryptophan 2-monooxygenases known from bacteria, such as iaaM or tms1 [19,20].

Indole glucosinolate biosynthesis mutants such as *sur1/rty*, and *sur2* are, on the other hand, characterized by strongly increased auxin contents and high-auxin phenotypes [21,22,23]. Mass spectrometric analyses demonstrated a significant increase in IAOx, IAM, and IAA in *sur1-1*, while indole-3-acetonitrile (IAN) levels appeared unchanged [10], which is suggestive for a redirection in the metabolic flux into the IAM pathway.

Increased IAM levels in *ami1* mutants have recently been shown to trigger abscisic acid (ABA) biosynthesis through the induction of *NCED3*, encoding for a key enzyme in the formation of ABA [24]. Along with the observation that IAM represses the expression of the K^+^ transporters *HAK/KT12* and *KUP4* that are assumed to contribute to elongation growth [25], this led to the hypothesis that the conversion of IAM to IAA catalyzed by IAM hydrolases represents a molecular nexus involved in the crosstalk between auxin and ABA. Although the transcriptomics analysis provided conclusive evidence for an intimate connection of AMI1-mediated IAM conversion and abiotic stress responses [24], the molecular mechanism by which changes in IAM levels are perceived and integrated remain elusive.

Here, we begin to address this open question by characterizing the impact of the simultaneous genetic blockade of the indole glucosinolate and IAM pathways in *A. thaliana*. By phenotypically and mass spectrometrically analyzing an *ami1 rty* double mutant, we demonstrate that accumulating IAM exerts a negative impact on embryo development. In addition, we report the comprehensive transcriptomics analysis of the mutant, which provided evidence for a number of physiological processes that are affected by the elevated IAM level in the mutant.

## 2. Results

### 2.1. The Introgression of the ami1-2 Mutation into rty1-1 Is Not Sufficient to Restore a Wild Type-Like Phenotype

Previous reports highlighted significant alterations in metabolite fluxes in indole glucosinolate biosynthesis mutants, including *sur1*/*rty*, and *sur2*. The experiments suggested an accumulation of IAOx and its further channeling into IAA [22,23,26,27,28]. While the *cyp79b2 cyp79b3* double mutant that is practically devoid of IAOx shows wild type IAA levels, downstream mutations in *sur1* translate into significantly increased IAOx, IAM, and IAA contents, suggesting the channeling of IAOx into IAA to proceed via IAM (Figure 1) [10]. The significantly elevated IAA levels in those mutants provoke strong auxin overproduction-related phenotypes, including epinasty of cotyledons and true leaves, significant amplification of lateral root numbers, and an increased root hair number and density [21,23].

Picking up on these lines of evidence, which suggest a purely auxin-mediated phenotype, we reasoned that a genetic block of the IAM pathway (IAOx→IAM→IAA) in indole glucosinolate mutants may restore a wild type (wt) phenotype, due to the impaired conversion of IAM to IAA. To this end, we introgressed the *ami1-2* mutant allele into the *rty1-1/+* genetic background, from which we expected to reduce the strong *rty* phenotype to the only moderate *ami1* phenotype [24]. In the F2 generation, we selected for plants homozygous for the *ami1-2* mutation using PCR genotyping. Three lines homozygous for *ami1-2* were identified and subjected to cDNA sequencing of the *RTY* gene (At2g20610), in order to genotype the zygosity state of the *rty1-1* mutation in these lines. *rty1-1* originates from an ethylmethane sulfonate (EMS) mutagenesis approach. Therefore, sequencing was necessary to genotype the state of the *rty1-1* mutation in the selected lines. As can be taken from Figure S1, two of the selected lines, line 5 and line 15, appeared to be homozygous *ami1 rty* double mutants. Remarkably, the identified *ami1 rty* mutants showed a dwarfish auxin-overproduction phenotype suggestive of a remaining conversion of IAM to IAA. (Figure S2).

**Figure 2:**
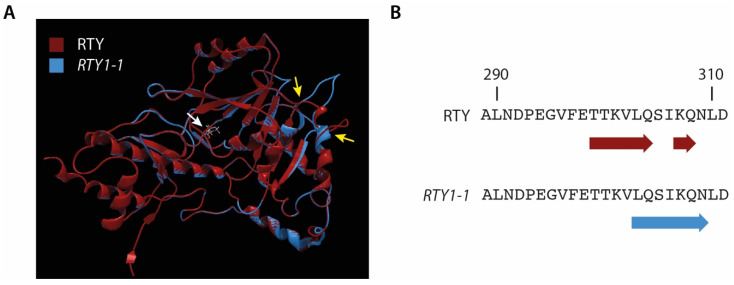
Modeling of structural changes in *RTY1-1* relative to RTY. (**A**) Overlay of the RTY (red) and *RTY1-1* (blue) 3D models. The loop section comprising the P213S mutation (white arrow) and the altered α-helical structure (yellow arrows) are indicated. (**B**) The panel additionally highlights the observed secondary structure changes that include alterations in an α-helical region at the C-terminal end of the protein.

At the protein level (Figure 2), the *RTY1-1* point mutation translates into a P213S amino acid exchange [29], which is likely to cause major structural changes in the RTY enzyme, which compromise its enzymatic activity. Protein modelling of RTY and *RTY1-1* against the 1.7 Å crystal structure of a closely related prephenate aminotransferase from Arabidopsis [30] (Protein Data Base (PDB): 6F5V) and subsequent structure predictions suggested the loss of one α-helix motif in *RTY1-1* (13 α-helices) relative to the wild-type protein (14 α-helices) in the C-terminal part of the protein. Notably, the P213S mutation is located in a loop region around 85 amino acids upstream of the estimated structural alteration. Hence, it might be concluded that the exchange of the non-polar proline to the polar serine residue leads to a displacement of the protein polarity, which ultimately changes the structure of the α-helical region.

As already mentioned, the *ami1 rty* double mutants still showed an unexpected auxin overproduction-related phenotype that implied a remaining conversion of IAM to IAA. However, this observation is in line with the recent identification of two additional acetamidase/formamidase family proteins described to act as IAM amidohydrolases in Arabidopsis [31]. Hence, it is plausible that the two IAM amidohydrolases, IAMH1 and IAMH2, are responsible for the remaining conversion of accumulating IAM to IAA. Interestingly, transcriptomics data from the presumably IAM accumulating *sur2* mutant and *iaaM* overexpressing tomato plants provide no indication for transcriptional alterations of the IAM amidohydrolase genes [32,33]. Along with the findings that IAM application triggers strong auxin-related root phenotypes in wild-type Arabidopsis and conditional *AMI1* overexpressor lines [24] and that Arabidopsis plants overexpressing the bacterial tryptophan 2-monooxygenase *iaaM* gene, producing IAM from l-Trp, also show considerable IAA and IAM overproduction [34,35], it must be concluded that Arabidopsis plants maintain a basal level of IAM amidohydrolases that readily convert accumulating IAM to IAA.

### 2.2. The ami1 rty Double Mutant Is Compromised in Germination

**Figure 3:**
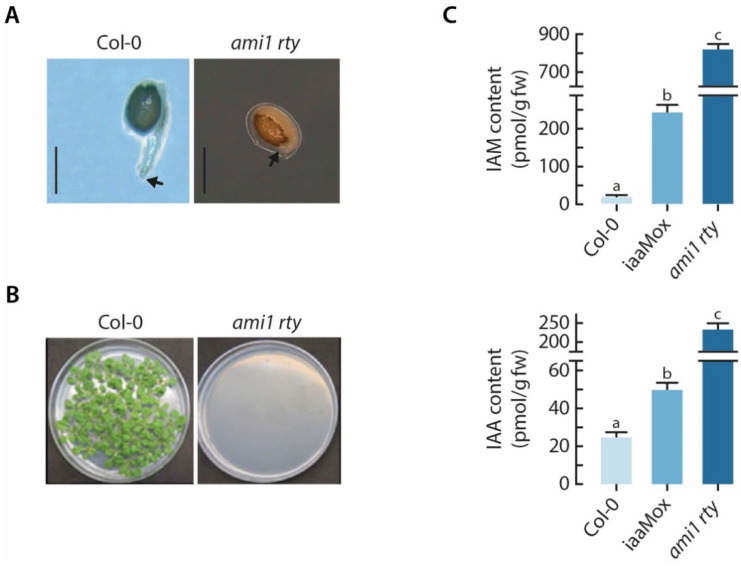
Seeds of *ami1 rty* mutants show a severe growth arrest and accumulation of IAM and IAA. (**A**) The photographs show close-ups of wild type (wt) (Col-0) and *ami1 rty* seeds grown on ½ Murashige & Skoog plates. The arrows mark the tip of the primary root two days after emergence. At this stage, the *ami1/rty* root stops growing. Scale bars = 1 mm. (**B**) The figure shows the comparison of wt (Col-0) and *ami1 rty* mutant seeds grown on ½ MS plates. The *ami1/rty* seeds abort germination, while wt (Col-0) seeds were able to germinate. (**C**) Analysis of IAM and IAA levels in wild-type Arabidopsis, iaaMox, and *ami1 rty*. Imbibed seeds from wild-type Arabidopsis (Col-0), iaaMox, and the *ami1 rty* double mutant were analyzed for their endogenous IAM (upper panel) and IAA levels (lower panel). The plots show mean values with their corresponding standard errors (SE), *n* = 5. To assess significant differences between the endogenous levels of each compound an analysis of variance (anova) of the scores followed by Tukey’s post-hoc test was performed. The different letters indicate significant differences, *p* ≤ 0.001.

After an additional round of selfing, we further analyzing the offspring of the two identified *ami1 rty* double mutants. The phenotypic assessment of the F3 generation revealed a severe impairment of the germination process for both lines. The obtained seeds did either not germinate at all or aborted germination after a very short period of time, ranging between 48 and 72 h, just after radicle protrusion through the seed coat (Figure 3).

The mass spectrometric analysis corroborated the high-auxin phenotype, providing evidence for a significant 10-fold increase in IAA levels in imbibed *ami1 rty* seeds compared to wt seeds. This underscores the notion that IAMH1 and IAMH2, respectively, contribute to the conversion of IAM to IAA in Arabidopsis. The iaaMox seeds, on the other hand, displayed an only two-fold increase in endogenous IAA levels relative to wt. With respect to the IAM contents, it became evident that *ami1 rty* seeds accumulate over 45 times the amount of IAM of wt seeds. In agreement with previously published data of IAM contents in conditionally *iaaM* overexpressing seedlings [35], the iaaMox seeds showed a significant accumulation of IAM (14 times). With respect to the data published for *sur1-1* [10] that reported a 34- and 21-fold increase in endogenous IAM and IAA levels, respectively, the presented data underscore the role of AMI1 in the conversion of IAM to IAA in Arabidopsis. Apparently, the introgression of *ami1-2* into the *rty1* indole glucosinolate biosynthesis mutant background considerably altered the conversion of IAM into IAA and led to an enhanced accumulation of IAM.

Auxin has been described to control seed dormancy in Arabidopsis through the interaction with ABA signaling in an ABI3-dependent manner [36]. Elevated IAA contents were shown to increase the dormancy in transgenic iaaMox seeds. In order to exclude dormancy effects through the significantly increased IAA level in the double mutant, we tried different stratification periods, ranging between 2 to 6 days, but under none of the tested conditions were we able to rescue *ami1 rty* mutant plants. However, given that most seeds were able to initiate germination, it must be concluded that seed dormancy is not the decisive factor responsible for the observed abortion of the germination process in the double mutants.

### 2.3. Alterations in Cellular IAA and IAM Contents Impact Embryo Size in Arabidopsis

**Figure 4:**
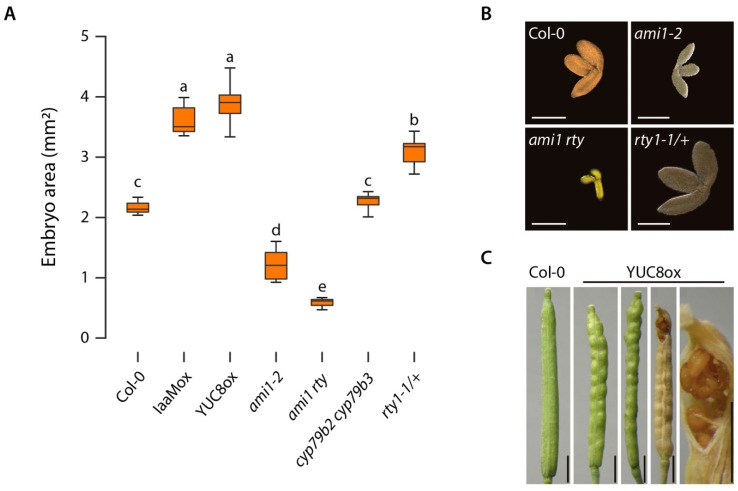
Effect of auxin on embryo and seed size in auxin mutants. (**A**) The box plots show the comparison of the embryo size of different auxin-related mutants. They display the median, quartiles, and extremes of the compared embryo sizes, *n* = 12. To assess significant differences in embryo size an analysis of variance (anova) of the scores followed by Tukey’s post-hoc test was performed. The different letters indicate significant differences, *p* ≤ 0.001. (**B**) Representative photographs of an *ami1-2*, *rty1-1/+*, and *ami1 rty* embryo in comparison to wt (Col-0). The photographs have the same scale, scale bars = 1 mm. (**C**) Representative pictures of siliques from Col-0 and YUC8ox plants. Scale bars = 5 mm.

It is widely accepted that auxin synthesized by fertilized achenes drives fleshy fruit development and ripening, e.g., in strawberries [37,38,39], and that genetic boosting of auxin formation in ovules through the tissue-specific expression of the *iaaM* gene promotes parthenocarpy in tobacco, eggplant, and tomato [40,41,42]. The role of auxin in regulating the development and maturation of dry dehiscent seeds, such as in the model plant Arabidopsis, is still less well understood, although great progress has been made over the past few years [43,44,45]. In previous studies, we observed that the overproduction of IAA in Arabidopsis significantly increased seed size, while the accumulation of IAM was shown to reduce it [24,25]. Auxin has also been demonstrated to significantly impact seed size and starch accumulation in pea [46]. In Arabidopsis, the embryo takes up large part of the space in the seed and, thus, greatly determines seed size, as the endosperm is reduced to a single cell layer [47]. In order to quantify the effect of altered IAA and IAM contents on seed development, we compared the embryo sizes of a number of auxin biosynthesis-related mutants in comparison to wt embryos (Figure 4).

As displayed in Figure 4, embryos of auxin overproducing YUC8ox and iaaMox lines [34,48] were significantly bigger than wt embryos, whereas the IAM accumulating *ami1* embryos [24] appeared to be considerably smaller. This confirmed our previous findings and highlighted the generally growth promoting effect of auxin in seed development. The effect on seed development in YUC8ox was so strong that the siliques were not able to follow the increased seed growth, which in consequence resulted in the premature opening of the valves of non-dehiscent siliques along the replum (Figure 4C). At the same time, our results suggested that either the excessive accumulation of IAM or the partially blocked conversion of IAM to IAA in the *ami1* mutant have growth inhibiting effects. Interestingly, the *rty* mutant [23,49], which is allelic to *superroot1* (*sur1*) [21], showed a slightly bigger embryo size than wild-type Arabidopsis. The *rty* seedlings are known to have elevated IAA contents [23], and can be expected to also have strongly elevated IAM levels, like *sur1* [10]. Taking the heterozygosity of the tested *rty1-1* seeds into account, the *rty1-1* embryos resemble those of iaaMox, that also contain elevated IAM and IAA levels [19,34]. The largely IAM-deficient *cyp79b2 cyp79b3* double mutant displayed no significant alteration of embryo size, which can likely be attributed to the wt IAA levels in this mutant [9,10,16]. Most remarkable, however, was the strong embryo phenotype of the *ami1 rty* double mutant. Potentiating the *ami1-2* embryo phenotype previously described [24], the *ami1 rty* double mutant embryos were to be significantly smaller than wt and *ami1-2* embryos (Figure 4A,B). Along with the abortion of the germination process, the strong *ami1 rty* embryo phenotype may be taken as an evidence that the production of viable seeds was considerably affected in the identified homozygous double mutant plants.

### 2.4. The ami1 rty Mutant Shows Major Impairments in Gene Transcription and Protein Synthesis

Considering the drastic curtailing of embryo growth in the *ami1 rty* double mutant and the detected significant accumulation of IAM in these mutant seeds, we concluded that the canalization of IAM into IAA during seed filling must be an important factor for proper seed development. It seems as if the excessive accumulation of IAM triggers limited embryo development, while the observed growth arrest of the *ami1 rty* mutants during the germination process is possibly attributable to the limitation of storage compounds in the seed. To address the question of what molecular processes are affected and responsible for the strong phenotype, we decided to take a comprehensive transcriptomics approach, comparing the transcriptional profile of imbibed *ami1 rty* mutant seeds with that of wt seeds by RNAseq. First, we analyzed the expression levels of 128 selected auxin metabolism-, signaling-, and transport-related genes in the *ami1 rty* mutant relative to wt (Table S1).

The directed assessment of target gene expression levels revealed that genes associated with auxin de novo-biosynthesis were not significantly altered in their transcription. This underpins that the high auxin contents in the mutant derive from the genetic block of indole glucosinolate biosynthesis and the reduced flux of IAM to IAA, and that alteration of de novo-auxin biosynthesis is not involved. At the same time, we found a significant induction of the Gretchen Hagen gene *GH3.5*. The *GH3.5* gene encodes an acyl amino synthetase that preferentially conjugates IAA to aspartate if IAA and Asp contents are high [50]. This may be rated as a response to counteract the high IAA contents in the mutant. With respect to the biosynthesis of l-Trp derived defense compounds, i.e., camalexin and indole glucosinolate, we were not able to find conclusive evidence for the redirection of the metabolic flux into the biosynthesis of camalexin. Consistent with the role of the significantly induced transcription factor *WRKY33* as a negative regulator of camalexin biosynthesis [15], we registered no induction of camalexin biosynthesis-related genes, such as *CYP71A13* and *PAD3*. Hence, it must be concluded that there is no alternative metabolic bypass and the entire metabolic flux normally directed into indole glucosinolate biosynthesis now passes through the IAM shunt. Additionally, we detected no significant changes in the expression of auxin transport-related genes, and only the *IAA9*, *IAA12/BODENLOS (BDL)*, and *PLETHORA5* (*PLT5*) transcriptional regulators appeared to be differentially expressed in the mutant. While the reduced expression of *IAA9* and *PLT5* may be neglected because of their only minor differential expression, the repression of *IAA12/BDL* was far more pronounced. IAA12/BDL is known to affect primary root formation and apical–basal patterning in embryos [51]. Aux/IAA auxin signaling repressor proteins act in response pairs with their particular auxin response factor (ARF) transcription factors. IAA12/BDL is described to closely interact with MONOPTEROS (ARF5) [52,53]. Hence, the transcriptional repression of *IAA12/BDL* may result in an increased amount of free ARF5, because of a reduced sequestration of the transcription factor by its transcriptional repressor. However, apical–basal pattern formation is apparently no issue in *ami1 rty*, as the embryos showed a clear polar organization (Figure 4B).

Since our directed approach did not disclose auxin-related processes that could explain the embryo and germination phenotype of *ami1 rty*, we next undertook an in-depth transcript profiling approach to obtain a broader picture of differentially expressed genes (DEGs) and their functional relationships. Employing an adjusted *p*-value (false discovery rate (FDR)) of < 0.05 and an arbitrarily chosen differential expression value of *log2FC* ≥ +1.5 for induced genes and *log2FC* ≤ −1.5 for repressed genes, respectively, 62 induced and 203 repressed DEGs were identified (see Table S1). To functionally score the DEGs, we used the MapMan software. The application returned a small number of key processes that are affected in the double mutant. These processes include RNA processing, ribosome assembly, nucleotide metabolism, translation, as well as protein modification and degradation. In addition, the results pointed towards the transcriptional alteration of stress response, signaling, and transport-related genes (see Table S1).

**Figure 5:**
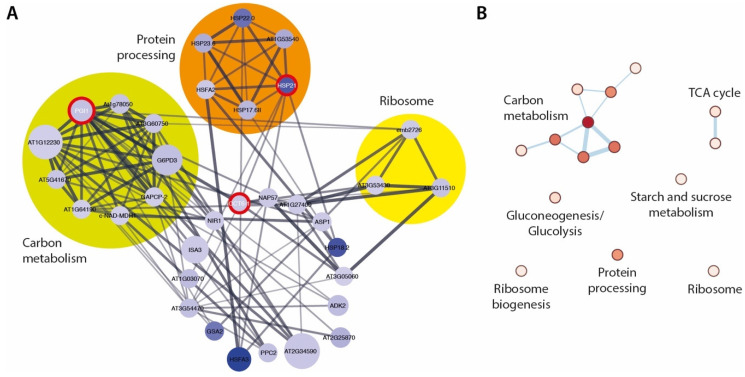
Network analysis of downregulated differentially expressed genes (DEGs) in *ami1 rty*. (**A**) Extracted network for the three DEGs with the highest betweenness centrality. The central hubs *CCT6-1*, *HSP21*, and *PGI1* are highlighted by red circles. The nodes are color coded according to the *log2FC* expression levels of the DEGs (white to blue), with dark blue marking the most repressed DEGs. The node size describes the false discovery rate (FDR) values, while the edge thickness reflects the betweenness score between the nodes. Densely connected nodes have been identified by using the Molecular Complex Detection (MCODE) clustering algorithm and are highlighted by colored circles. (**B**) Kyoto Encyclopedia of Genes and Genomes (KEGG) pathway enrichment analysis of the sub-network extracted for *CCT6-1*, *HSP21*, and *PGI1*. The color (white to red) gives account on the normalized enrichment score of the corresponding KEGG pathway.

With the aim to gain more detailed insight into the relationships of the selected DEGs, we performed a functional association network analysis using the stringApp in Cytoscape. For the induced DEGs, we obtained a network with 57 nodes and 21 edges. Further analysis of the network, focusing on the node with the highest degree of connectivity and betweenness centrality [54], *SMALL DEFENSE-ASSOCIATED PROTEIN 1 (SDA1)*, provided a subnet with 7 nodes and 12 edges. Along with the genes *ZAT11* and *CML37*, the central *SDA1* hub could be associated with oxidative stress responses [55]. For the downregulated DEGs, we inferred a network with 198 nodes and 422 edges. The gene with the highest degree of connectivity was the ribosome biogenesis related *FIBRILLARIN 2* (*FIB2*) gene [56] with 27 connections, followed by the ribosomal L22p/L17e family protein gene *At1g27400* with 25 connections. FIB2 is a particularly interesting candidate, because it directs a requisite step in rRNA processing and ribosome assembly [57]. However, from a more general perspective, the network analysis pointed towards an impairment of ribosome-dependent processes, which confirms the previously discussed classification of the DEGs. The Kyoto Encyclopedia of Genes and Genomes (KEGG) [58] pathway enrichment analysis of the two mentioned sub-networks also highlighted ribosome- and ribosome biogenesis-related processes to be underrepresented in the *ami1 rty* mutant (Figure S3). Among the downregulated DEGs, the genes of a TCP-1/cpn60 chaperonin family protein *CCT6-1*, *HEAT SHOCK PROTEIN 21* (*HSP21*), and *PHOSPHOGLUCOSE ISOMERASE 1* (*PGI1*) showed the highest betweenness centrality. On the basis of the topology of the extracted network for the three central hubs, we performed a KEGG pathway enrichment analysis. As can be taken from Figure 5, the network inferred from the betweenness centrality in combination with a projection onto the KEGG pathway maps identified a series of metabolic pathways, including ribosome biogenesis, amino acid biosynthesis, and carbon metabolism pathways, that are seemingly affected in the *ami1 rty* double mutant.

**Figure 6:**
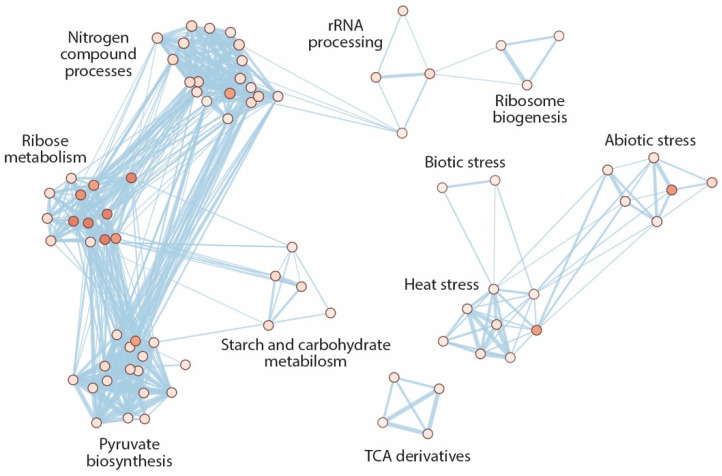
Gene Ontology (GO) term enrichment map for downregulated DEGs. GO terms that share members are shown in connected clusters. Cluster labels were retrieved using the AutoAnnotate v1.3.3 application in Cytoscape. The node color intensity reflects the normalized enrichment score (NES) of the underrepresented terms (white to red) with red marking the highest NES. The node size gives account on the number of members in the particular GO term.

Gene Ontology (GO) biological process terms represent a rich resource for the functional characterization of large “omics” data sets. GO annotations include a mix of manually curated and electronically inferred sources [59]. Taking advantage of this resource, we next subjected the previously derived functional association networks for both up- and downregulated DEGs to a GO enrichment analysis. However, for the upregulated DEGs we were not able identify enriched GO term classifications. As demonstrated in Figure 6, the inferred GO enrichment map largely confirmed the previously obtained results, highlighting a significant underrepresentation of biological processes related with translational processes, including ribosome biogenesis and assembly, rRNA processing, and ribonucleotide biosynthesis and carbohydrate metabolism. Most notably, the study brought additional insight into the underrepresentation of GO classifications associated with plant stress responses, as well as responses to chitin. Moreover, it highlighted the impairment of the temperature stress response in *ami1 rty* (Figure 6).

With respect to the repressed genes falling into the plant stress response clusters, the small heat shock protein genes *HSP18.2, HSP17.6II*, and *HSP21*, which contribute to stabilize translation factors and are involved in conferring desiccation tolerance [60], and the heat shock transcription factors *HSFA2* and *HSFA3* that are involved in the control of thermo- and osmotic stress tolerance [61,62] have to be mentioned.

### 2.5. Hyperaccumulation of IAM Provokes Repression of Elongation Growth Regulatory Pathways

**Figure 7:**
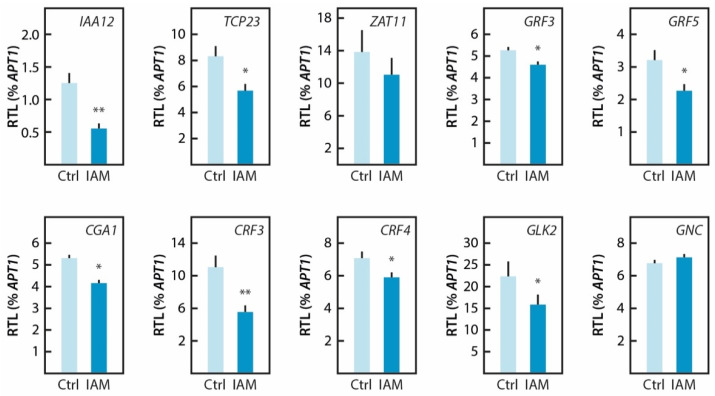
Validation of RNAseq data by qRT-PCR. The figure shows the relative transcript levels (RTL) of selected target genes (arithmetic mean ± SE, *n* = 9) normalized to *APT1* as a constitutively expressed control gene in 7-day-old wt (Col-0) seedlings. Before RNA extraction, the seedlings were either treated for 2 h with 20 µM IAM (IAM) or with a control solution (Ctrl) free of IAM. The selected target genes were: *IAA12/BDL*, *Aux/IAA protein 12* (At1g04550); *TCP23*, *TEOSINTE BRANCHED 1/CYCLOIDEA/PROLIFERATING CELL FACTOR 23* (At1g35560); *ZAT11*, *ZINC FINGER TRANSCRIPTION FACTOR 11* (At2g37430); *GRF3*, *GROWTH REGULATING FACTOR 3* (At2g36400); *GRF5*, *GROWTH REGULATING FACTOR 5* (At3g13960); *CGA1*, *CYTOKININ-RESPONSIVE GATA FACTOR 1* (At4g26150); CRF3, *CYTOKININ RESPONSE FACTOR 3* (At5g53290); *CRF4*, *CYTOKININ RESPONSE FACTOR 4* (At4g27950); *GLK2*, *GOLDEN-LIKE 2* (At5g44190), and *GNC*, *GATA, NITRATE-INDUCIBLE, CARBON-METABOLISM INVOLVED* (At5g56860), in order of appearance. Asterisks indicate significant differences between the corresponding control sample and the IAM treated sample. (Student’s *t*-test; * *p* ≤ 0.05, ** *p* ≤ 0.01).

In order to explore gene regulatory networks that are possibly involved in causing the *ami1 rty* embryo phenotype and to experimentally validate the conclusions drawn from the RNAseq experiment, we centered our interest on transcription factors with significantly altered expression levels. A compilation of these factors can be found in Table S1. Overall, 19 transcription factors were identified, from which three were significantly induced, while 16 appeared to be significantly repressed. As presented in Figure 7, ten of these genes were selected and their transcriptional response to a short-term treatment with IAM was tested by qRT-PCR.

The most pronounced induction was registered for the *MYB4* gene, followed by *ZAT11*. MYB4 is a R2R3-subfamily MYB domain protein associated with radiation and thermal stress responses in Arabidopsis [63,64]. The induced Cis_2_/His_2_-type zinc finger proteins ZAT7 and ZAT11 are also associated with abiotic stress, particularly with responses to salinity stress and nickel tolerance, respectively [65,66]. Most interesting, however, was the identification of the ABA response-related transcriptional regulators GOLDEN2-LIKE 1 and −2 (GLK1/2) that are reported to be involved in controlling seed development [67]. The finding is particularly interesting because increased IAM contents are known to trigger ABA biosynthesis [24] and *GLK2* was registered among the repressed factors.

Among the repressed transcription factors, we also found several stress related targets, such as the aforementioned heat shock factors *HSFA2* and *−3* [68,69], as well as the cytokinin response factor *CRF3* involved in cold stress responses and freezing tolerance in plants [70]. This further substantiates the tight connection between IAM accumulation and plant stress responses. In addition, we identified the significantly repressed *TEOSINTE BRANCHED 1/CYCLOIDEA/PROLIFERATING CELL FACTOR* genes *TCP10* and *TCP23* as potential molecular targets. TCPs are reported to play pivotal roles in the control of morphogenesis of shoot organs and developmental processes, such as flowering [71,72]. Remarkably, however, is the direct connection of TCP10 with auxin homeostasis-related processes. Especially the downregulation of TCP10 in the *iamt1-D* gain-of-function mutant [73] implies the existence of regulatory loops that connect developmental processes with endogenous auxin levels, which possibly involve the HD-ZIP factor HAT3 that might contribute to the control of cotyledon development [74], and that has also been registered among the repressed factors.

However, two other groups of transcription factors, including the gibberellin signaling repressors *GNC* and *GNL/CGA1* [75], and the growth-regulating factors *GRF3* and *GRF5* that are recognized as mediators of organ size establishment and cell proliferation [76,77], attracted our interest, as they promised mechanistic insight into the crosstalk between IAM and gibberellin, as well as with general growth control.

In order to investigate as to whether IAM can trigger similar responses in wild-type Arabidopsis, we tested the gene regulatory effect of exogenously applied IAM in 7-days-old wild-type seedlings. With the rationale to avoid problems regarding the permeability of IAM through the seed coat, we decided to employ young seedlings instead of seeds. Moreover, our previous work demonstrated that IAM contents in imbibed seeds are high and drop sharply within the first 3–4 days of germination [78], which correlates with the expression pattern of AMI1 [24]. By using slightly older seedlings, we tried to assure that the already high IAM in the seeds does not interfere with the exogenously applied IAM.

Apart from *ZAT11*, for which the induction could not be confirmed, we studied nine further genes identified as repressed in *ami1 rty*. Apart from *GNC*, the repression of all other selected genes by IAM could be confirmed by qRT-PCR analysis. However, it has to be remarked that the impact of the short-term IAM treatment is generally less pronounced as in the *ami1 rty* double mutant, which is characterized by constantly elevated endogenous IAM levels.

## 3. Discussion

Auxins are well-characterized phytohormones that control a huge variety of different growth- and development-related processes. The role of auxin in pattern formation and embryogenesis has been studied in great detail [79,80]. Several recent publications ascribe auxin also an important role in later stages of seed development, including seed filling, dormancy control, and germination. An example is the pivotal role of auxin biosynthesis in the endosperm for proper seed coat formation [81], the increased dormancy of IAA overproducing mutant plants [36], or the observed impact of reduced auxin formation on seed size growth and starch formation in legumes [46]. A similar positive regulatory relationship of auxin formation and starch production in rice has also been suggested [82]. On the other hand, nutrient allocation and plant hormone crosstalk are assumed to play essential roles in seed development as well. A number of K^+^ channels and transporters are downstream targets of auxin [25], and auxin biosynthesis is reported to be controlled by sugars [83,84]. Most recent results give reason to speculate on an involvement of auxin–ABA crosstalk in this process [85,86]. However, overall, the role of auxin in orchestrating seed development has received only little attention, despite is undeniable role as key regulator of plant development. A more detailed analysis may entail biotechnological advances that could be harnessed to improve agricultural productivity in an environmentally friendly manner.

Our previous work shed some light on the role of IAA and its direct precursor, IAM, in seed development and a connection with phytohormone crosstalk [24,25,48,87]. Our findings led to the hypothesis that IAM is a negative plant growth regulator and that the enzymatic conversion of IAM to IAA by AMI1 terminates its growth inhibitory action. A similar function of another amidase signature family member, FATTY ACID AMIDE HYDROLASE (FAAH), has already been demonstrated. FAAH catalyzes the hydrolysis of *N*-acylethanolamines, which represent lipid signaling molecules, thereby controlling their action [88,89]. In this study, we aimed at addressing the question on the molecular and physiological consequences of endogenously accumulating IAM contents in Arabidopsis. For this reason, we decided to cross the indole glucosinolate biosynthesis mutant *rty1-1*, which has significantly elevated IAM and IAA levels, with the *ami1-2* mutant, to block the enzymatic conversion of IAM to IAA. Against our initial expectation, homozygous double mutants showed no *ami1*-like phenotype and IAA levels, but resembled a mild *rty* phenotype (Figure S2) and were characterized by elevated IAA contents (Figure 3). However, the observed remaining IAM hydrolase activity confirmed our previous observation of a remaining amidase activity in *ami1* mutants [24]. Moreover, it is in line with the recent identification of two additional enzymes, IAMH1 and IAMH2, that also contribute to the conversion of IAM to IAA in Arabidopsis [31]. Taking the reported remaining 40 to 45% IAM hydrolase activity of *ami1* mutants into account, the obtained data neatly reflect the loss of AMI1 activity in the mutant, particularly because only one of the two other amidases, *IAMH1*, shows expression during seed development [90].

To our surprise, seeds of the offspring of homozygous *ami1 rty* mutant plants were nonviable. They aborted germination short after the radicle broke through the seed coat. Given that the homozygous parent plants germinated normally and set a small number of siliques, it must be concluded that they were derived from ancestors heterozygous for *rty*, and that, e.g., the nutrient acquisition during seed filling is compromised in the homozygous parents, which may explain the impairment of germination in the offspring. To gain closer insight into the embryo phenotype, we inspected the *ami1 rty* embryos and compared them to a series of other auxin biosynthesis-related mutant embryos. The quantitative assessment of the embryos disclosed a striking reduction in the size of *ami1 rty* mutant seeds. The phenotypic inspection was accompanied by the mass spectrometric analysis of IAM and IAA contents in imbibed seeds. In comparison to the absolute IAM:IAA ratio of 0.7:1 in wt and the reported increase to a 1.6:1 ratio in the *sur1-1* mutant [10] that is allelic to *rty*, we found an increase in the IAM:IAA ratio to 4.9:1 in double mutant seeds. From this, we conclude that the embryo phenotype of *ami1 rty* is most likely attributable to the further elevated IAM contents. To obtain additional insight into the role of IAM catabolism on seed development, it will be highly interesting to generate an *ami1 iamh1 iamh2* triple mutant in the future, although such mutants might be prone to nonviability.

With the objective to further our understanding about the molecular consequences of the observed IAM accumulation in the dwarfish *ami1 rty* mutant embryos, we subjected double mutant seeds to whole-genome transcript sequencing (RNAseq) and compared the obtained transcriptional profile with that of wild-type seeds. In first place, we checked the expression of a subgroup of 128 auxin-related genes. The targeted analysis provided no evidence for the misregulation of auxin metabolism-, transport-, or signaling-related genes. In particular, the missing activation of genes of the camalexin biosynthesis pathway is noteworthy, because we initially speculated that the accumulation of IAOx or IAM may trigger a metabolic redirection into the camalexin pathway. On the contrary, we found the induction of *WRKY33*, a transcriptional repressor of camalexin biosynthesis (Table S1). The mRNA sequencing revealed, however, a significant downregulation of translation-related genes. The primarily affected processes included ribosome biogenesis and assembly, as well as rRNA processing (Figure 6 and Table S1). A directed search for the differential expression of master regulators of ribosome biogenesis, such as *SMO4* (*SMALL ORGAN 4*) or *MAS2* (*MORPHOLOGY OF ARGONAUTE 1-52 SUPPRESSED 2*) [91,92], was however not successful. In addition, our network analysis of the expression data revealed that the *ami1 rty* mutant seeds fall significantly short in the expression of sugar and amino acid metabolism-associated transcripts. Considering the dwarfish embryo phenotype and impaired germination, we conclude that *ami1 rty* mutants are most likely compromised in seed filling. The major storage organ in Arabidopsis seeds are the cotyledons. The substantially reduced cotyledon size in the double mutant is suggested to prevent the deposition of adequate storage reserves (oil, protein, starch) in the seed, which ultimately leaves the germinating seedling with insufficient energy resources to cover the initial growth phase until the seedling establishes an autotroph lifestyle.

The transcriptomics analysis provided additional evidence for the differential regulation of thermal- and oxidative stress related genes (Figure 6). This observation matches well with our previous finding of an intimate connection between abiotic stress responses and increased IAM contents [24]. The misregulation of a substantial number of small heat shock protein genes and the involvement of drought stress related processes additionally point towards an important misregulation of the establishment of desiccation tolerance related processes. Of particular interest for our future work was, however, the identification of a number of plant growth regulating processes that were significantly repressed in the *ami1 rty* double mutant. The repression of the growth-regulating factors *GRF3* and *GRF5* in IAM treated wild-type Arabidopsis seedlings confirmed a direct connection between IAM and the regulation of plant growth. In addition, our bioinformatic analyses brought a small number of plant hormone-regulatory circuits to light, which suggest an even more important role of IAM in plant hormone crosstalk, connecting with gibberellin signaling through the repression of *GNC* and *CGA1*, two transcription factors that act downstream of the DELLAs on gibberellin signaling [75], although the transcriptional repression by IAM was only confirmed for *CGA1*. The identification of the repression of two TCP family members, *TCP10* and *TCP23*, that are assumed to play pivotal roles in the control of shoot morphogenesis and developmental transitions, such as flowering, was also interesting [71,72]. A significant repression of *TCP23* in wild type seedlings by IAM could be confirmed by transcript quantification.

The misregulation of these transcription factors is possibly a reason for the abortion of *ami1 rty* seedling development. The question on the molecular mechanism by which embryo development and seed filling, including the transfer of storage compounds from maternal tissues to the seed, are affected in *ami1 rty*, cannot be conclusively answered from our RNAseq results. The studied transcriptional profiles provide evidence on early germination-related processes, but do not allow for coherent conclusions on processes that occur during embryogenesis and seed maturation. However, considering the substantial impairment of carbohydrate metabolism-, ribosome biogenesis-, and amino acid biosynthesis-related processes, our interpretation is that the increased accumulation of IAM during seed development interferes with the remobilization of carbohydrates and nitrogen-containing compounds from maternal tissues, which in consequence compromises seed development and finally results in the production of nonviable seeds. The detailed investigation and elucidation of IAM triggered processes and how IAM signaling is integrated will be a particularly thrilling task for our future work.

## 4. Materials and Methods

### 4.1. Plant Material and Growth Conditions

The presented experiments used the *Arabidopsis thaliana* Col-0 background (N1092) as reference. The *rooty* mutant (*rty1-1*/+, stock N8156) was obtained from the Nottingham Arabidopsis Stock Center (NASC). The mutants iaaMox [19,93], YUC8ox [48], *ami1-2* [24], and *cyp79b2 cyp79b3* [16] have previously been described in close detail. The *iaaMox* mutant [93] was kindly provided by Dr. Yunde Zhao. The *ami1 rty* double mutant was generated by crossing the *ami1-2* mutant with *rty1-1/+* plants, followed by geno- and phenotyping of the offspring in the F2 and F3 generation, respectively.

Seedlings were grown under sterile conditions on solidified ½ Murashige & Skoog-medium containing 1% (*w/v*) sucrose in Petri dishes [94]; plantlets were kept under constant environmental short day conditions (8 h light at 24 °C, 16 h darkness at 20 °C, photosynthetically active radiation 105 µmol photons m^−2^ s^−1^ from standard white fluorescent tubes) for two to three weeks. Thereafter, plants were transferred to a mixture of soil and sand (2:1) and kept under long day conditions (16-h photoperiod) in a greenhouse, which was maintained under constant climatic conditions, 22 to 24 °C during daytime and 18 to 20 °C overnight. The photosynthetically active radiation was no less than 150 µmol photons m^−2^ s^−1^ (supplementary light, if required, was provided from sodium-vapor lamps).

### 4.2. Genotyping of the Ami1 Rty Double Mutant

To genotype *ami1 rty* mutants, a two-step approach was taken. First, the zygosity state of the *ami1-2* T-DNA integration was analyzed by PCR [95]. Instead of running a multiplex PCR with the two *AMI1*-specific primers Pr1/Pr2 and the T-DNA-specific primer Lb, we performed three independent reactions (Pr1/Pr2, Pr1/Lb, Pr2/Lb) for each selected line. After identifying lines homozygous for the *ami1-2* mutation, we extracted total RNA [96] and prepared cDNA libraries from those lines using M-MLV reverse transcriptase and oligo(dT)_15_ primer according to the manufacturer’s instructions. Next, we amplified the *RTY* gene using the primers RTY-fwd and RTY-rev by PCR. (See Table S2) for primer sequences. The obtained *RTY* PCR fragments of the three lines were then sequenced on an ABI 3730 xl sequencer by the company Stabvida (http://www.stabvida.com (accessed on 30 November 2020)). Sequence and trace file analysis were carried out using the CLC Main Workbench 7 (Qiagen).

### 4.3. Modelling of the RTY and RTY1-1 Protein Structure

The three-dimensional structures of RTY and *RTY1-1* were modeled by using the SWISS-MODEL interface (http://swissmodel.expasy.org (accessed on 30 November 2020)) [97], utilizing the 1.7 Å crystal structure of a bifunctional aspartate aminotransferase and glutamate/aspartate-prephenate aminotransferase (PAT, At2g22250) deposited in the Research Collaboratory for Structural Bioinformatics (RCSB) Protein Data Base (PDB: 6F5V) [30]. In accordance with [29], we used the PAT structure as a template, although RTY is structurally closely related with alanine aminotransferases, e.g., from *Hordeum vulgare* (PDB: 3TCM), because this crystal structure originates from the same species. Structural examination was performed using either PyMOL v1.47 (http://pymol.org (accessed on 30 November 2020)) or CLC Main Workbench 7.

### 4.4. Quantitative Comparison of Embryo Sizes

Dry seeds from wt Arabidopsis and the different auxin mutant lines were surface sterilized using successive treatments with 70% ethanol (*v/v*, 5 min) and a 5-7% sodium hypochlorite solution (*v/v*, 5 min), before being rinsed three times with water. Afterwards, the seeds were left overnight at 4 °C in diethyl pyrocarbonate (DEPC) water. Under a binocular (Leica MZ10 F), seeds were then transferred onto an object slide and mixed with glycerol. After removing the seed coat from the embryo, pictures were taken with 25-fold magnification using a color CCD camera (Leica DFC 420C, 5 Mpixels). Subsequently, the embryo size was measured by determining the embryo area employing the imageJ software [98].

### 4.5. Mass Spectrometric Analysis of IAA and IAM

Endogenous IAM and IAA contents were analyzed by liquid chromatography-mass spectrometry following a previously published protocol [24]. In brief, organic compounds were extracted from 100 mg of imbibed seeds into 1 mL ice-cold potassium phosphate buffer (50 mM, pH 7.0) containing 1% diethyldithiocarbamic acid sodium salt and 50 pmol of [^2^H_5_,^15^N]-IAM and [^2^H_5_]-IAA as internal standards. The organic phase was transferred into fresh tubes and acidified (pH 2.7) by adding 1 M hydrochloric acid. After the samples were pre-purified using 1 mL HLB columns, the eluates were dried in a vacuum and re-dissolved in 45 µl methanol with 0.1% formic acid (*v/v*). Subsequently, 10 µL of the samples were subjected LC-MS analysis employing an Ultimate3000 RSLC system (Dionex) and a microTOF-Q II mass spectrometer (Bruker Daltonics). In order to determine the analyte contents, the following ion transitions were monitored: IAM, m/z = 175.2 130.1; [^2^H_5_,^15^N]-IAM, m/z = 181.2 135.1 (retention time, 6.3 min); IAA, m/z = 176.2 130.1; [^2^H_5_]-IAA, m/z = 181.2 135.1 (retention time 8.8 min). To integrate the corresponding peak areas, the extracted ion chromatograms were analyzed using the DataAnalysis software package (Bruker Daltonics).

### 4.6. Transcriptomics Analysis of the Ami1 Rty Double Mutant

Total RNA from 100 mg imbibed Arabidopsis wt and *ami1 rty* mutant seeds was extracted using the RNasy Plant Mini Kit (Qiagen) according to the manufacturer’s instructions. The quality and quantity of the extracted RNA was tested by absorbance analysis using a Nanodrop^®^ ND-1000 spectrophotometer (ThermoFisher). Additionally, the RNA samples were tested on a Bioanalyzer 2100 (Agilent) by the CNB Genomics Service (Madrid). Library construction and RNA sequencing (SE50) was performed by the Beijing Genomics Institute (BGI) on Illumina HiSeq™ 2000 machines. Basic data analysis, including data filtering, sequence alignment [99], transcript quantification [100], and differential gene expression analysis [101] was also performed by BGI. For each genotype three biological replicates were processed. The resulting *p*-values were adjusted for multiple testing using the Benjamini–Hochberg correction [102]. An adjusted *p*-value (FDR) of <0.05 and absolute differential expression of *log2FC* ≥ 1.5 were arbitrarily chosen to select differentially expressed genes (DEGs).

The functional classification of DEGs was performed using the MapMan v3.6 software [103], paying special attention to DEGs related with transcriptional regulation and development. Furthermore, functional relationships between the DEGs were investigated employing the stringApp v1.3 [104], MCODE v2.0 [105], and EnrichmentMaps v3.3.1 [106] in Cytoscape v3.8.2 [107]. In order to analyze the importance of the nodes in the inferred networks, the nodes with the highest degree of connectivity (k) and betweenness centrality (BC) were examined in closer detail.

Selected transcripts were validated in independent experiments by qRT-PCR. For this, 7-day-old wt seedlings were incubated for 2 h either in ½ MS or ½ MS with 20 µM IAM. For each condition, total RNA from three independent biological replicates was harvested and analyzed in triplicate (technical replicates). First-strand synthesis was performed according to the supplier’s instructions, using M-MLV reverse transcriptase and oligo(dT)_15_ primer (Promega). Two nanograms of cDNA were used as template for the qRT-PCR, which was performed according to the manufacturer’s instructions using the FastStart SYBR Green Master solution (Roche Diagnostics) on a Lightcycler 480 Real-Time PCR system (Roche Diagnostics). Relative quantification of expression was calculated after data analysis by the Lightcycler 480 software (Roche Diagnostics), using the comparative 2^−ΔΔCT^ method [108] with *APT1* (At1g27450) as the reference gene [109]. See Table S2 for primer sequences.

### 4.7. Statistical Analysis

We used JASP v0.14.1 for statistical data assessment and the generation of plots. The box plots display the median, quartiles, and extremes of the compared embryo sizes. One-way anova and Tukey’s post-hoc test or Student’s *t*-test were performed to statistically analyze the data. Sample sizes (n) for each experiment are given in the respective figure legends.

## Data Availability

All data supporting the findings of this study are available within the paper and within its supplementary data published online.

## Supplementary Materials

The following supplementary material is available online at https://www.mdpi.com/1422-0067/22/4/2040/s1, Figure S1: Genotyping of the ami1 rty double mutant, Figure S2: Phenotype of mutants used in this study, Figure S3: EnrichmentMap and GO term enrichment analyses of identified DEGs in ami1 rty, Table S1: Differential gene expression analysis of ami1 rty, Table S2: Primers used in this study.

## Abbreviations

cDNA	complementary DNA
DEPC	diethyl pyrocarbonate
FDR	false discovery rate
FC	fold-change
qRT-PCR	quantitative reverse transcriptase polymerase chain reaction
T-DNA	transfer DNA
