# Supplementary material for: Accumulation of the Auxin Precursor Indole-3-Acetamide Curtails Growth through the Repression of Ribosome-Biogenesis and Development-Related Transcriptional Networks
Source: Int J Mol Sci. 2021 Feb 18;22(4):2040. doi: 10.3390/ijms22042040 (PMC7923163; doi:10.3390/ijms22042040)
Supplement: Supplementary file 1 [file ijms-22-02040-s001.zip › Supplementary/Supplementary Figures.docx]

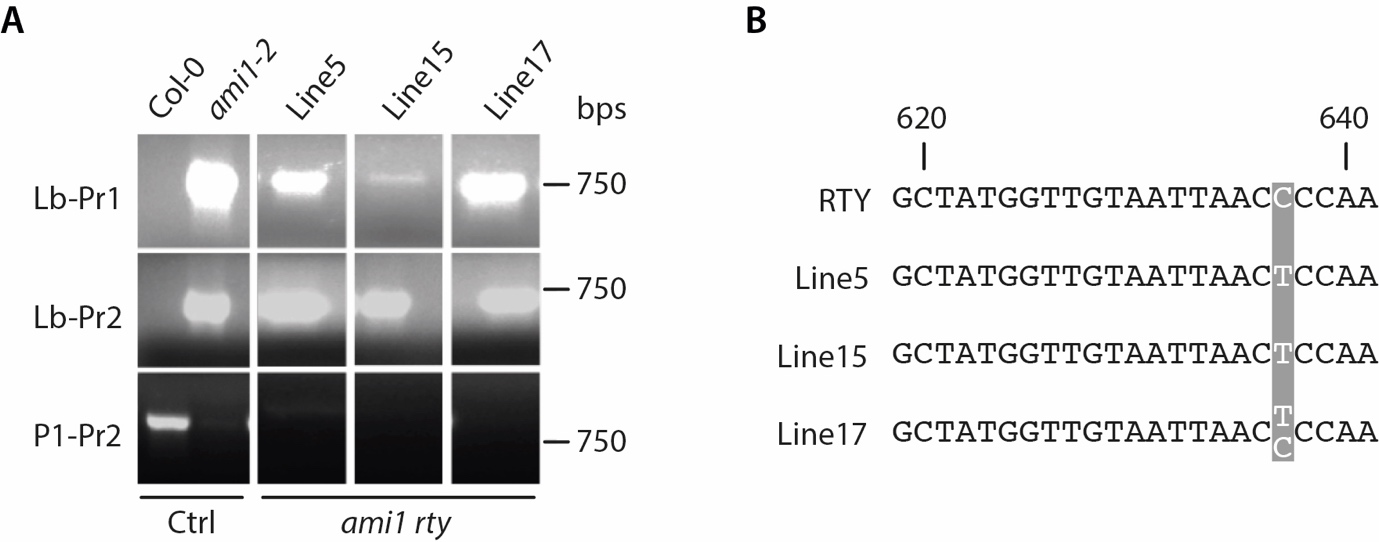


**Figure S1.** Genotyping of *ami1/rty* mutants. **(A)** PCR analysis comparing three selected double mutant lines with wt (Col-0) and *ami1-2* control (Ctrl) seedlings. The figure shows the results of three individual PCR reactions using either a T-DNA specific primer (Lb) and a 5’ *AMI1*-specific primer (Pr1), a T-DNA specific primer (Lb) and a 3’ *AMI1*-specific primer (Pr2) or the two *AMI1*-specific primers, Pr1 and Pr2. **(B)** Section of the alignment of the annotated *RTY* cDNA with the sequences obtained from the sequencing of three selected mutants, homozygous for the *ami1-2* mutation. The figure shows the point mutations at position 637 in lines 5 and 15 (grey box). The analysis of the trace files of line 17 revealed the heterozygosity of this line. Nucleic acid positions in the cDNA section are indicated.


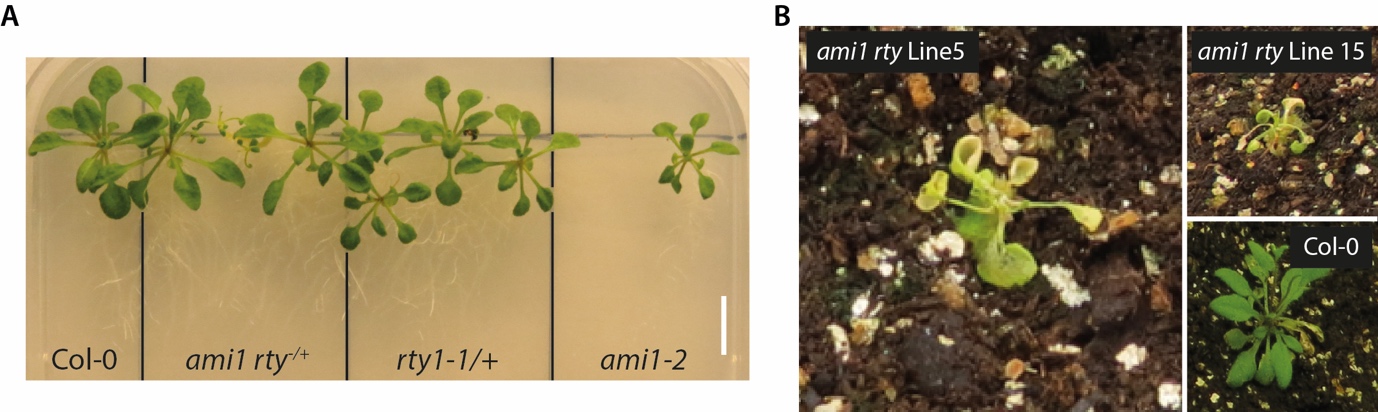


**Figure S2.** Phenotype of mutants used in this study. **(A)** Comparison of wild-type Arabidopsis (Col-0) with the parental plants *rty1-1/+* (♀), and *ami1-2* (♂), as well as the crossed double mutant *ami1 rty^-/+^* heterozygous for the *rty1-1* mutation. **(B)** Representative pictures of the phenotype of obtained *ami1 rty* double mutants in comparison to Col-0 plants.


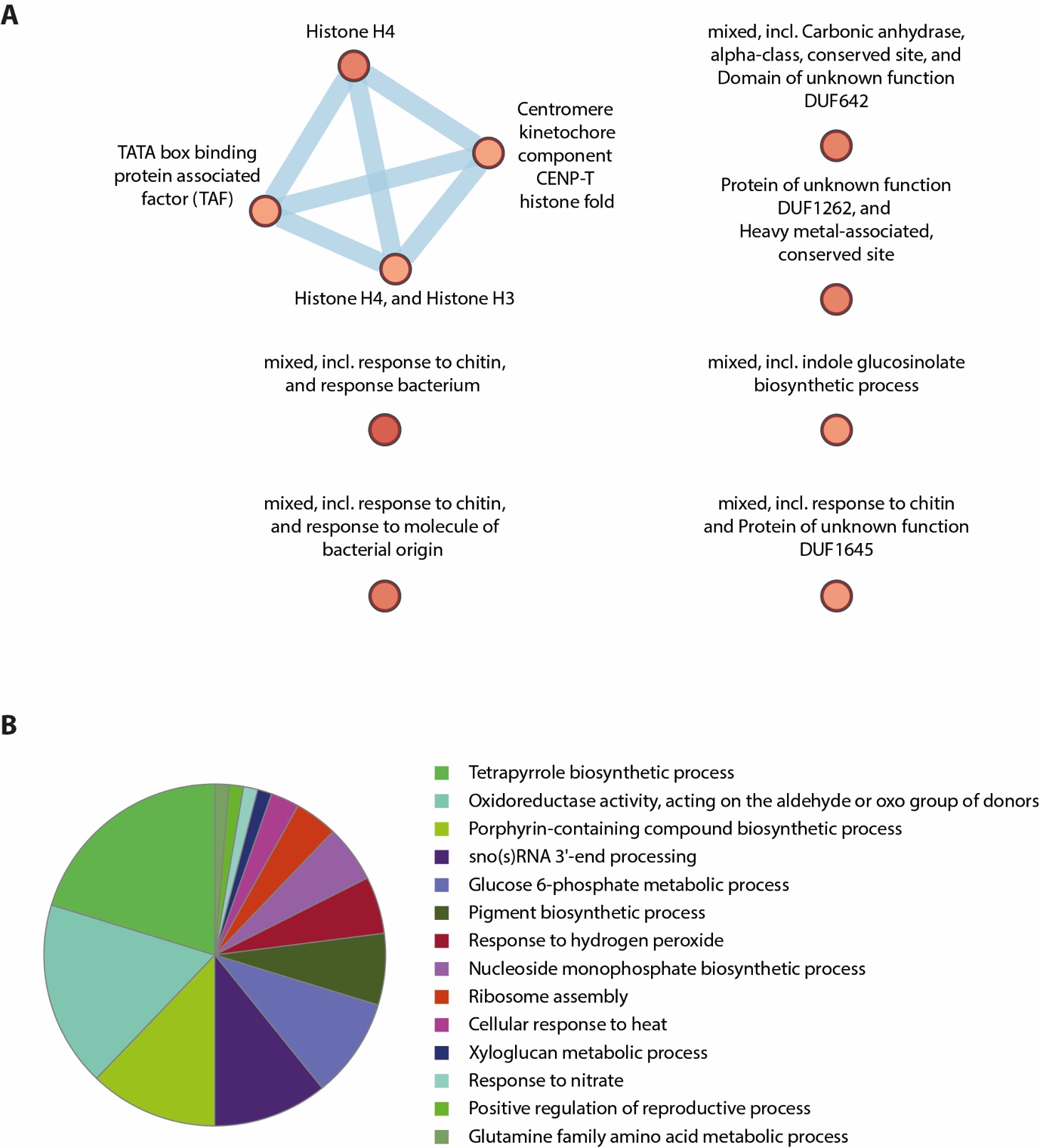


**Figure S3.** EnrichmentMap and GO term enrichment analyses of identified DEGs in *ami1 rty*. **(A)** EnrichmentMap analysis for the 62 induced induced DEGs. **(B)** Metabolism catalogs of enriched biological functions for downregulated genes in *ami1 rty* according to GO classifications.
